# Plasma-activated water irrigation increases mortality of immature spider mites (*Tetranychus urticae*) on tomato plants

**DOI:** 10.1038/s41598-025-05629-2

**Published:** 2025-07-01

**Authors:** Patrice Jacob Savi, Sydney Robertson, Anil Mantri, Bruno Augusto Mattar Carciofi, George Amponsah Annor, Christian Nansen

**Affiliations:** 1 Department of Entomology and Nematology, University of California, Davis, CA 95616 USA; 2 Department of Biological and Agricultural Engineering, University of California, Davis, CA 95616 USA; 3 Department of Food Science and Nutrition, University of Minnesota, Saint Paul, MN 55455 USA

**Keywords:** Life table parameters, Two-sex life table analysis, Plant resistance, Innovative pest management, Sustainable pest management, Biotechnology, Developmental biology, Ecology, Entomology

## Abstract

Supplementary irrigation with plasma-activated water (PAW) has been shown to boost seed germination and seedling vigor, and it has the potential to induce host plant resistance against pest populations, such as, two-spotted spider mites (TSSM) (*Tetranychus urticae* Koch). However, there is limited knowledge about the relative susceptibility of TSSM life stages to supplementary PAW irrigation. Here, we used age two-sex life table analysis to examine demographic parameters on leaf discs from control tomato plants (no PAW irrigation) and from plants receiving supplementary PAW irrigation, PAW1 and PAW2 (treatment of water for 6.0 and 9.4 min with atmospheric plasma jet respectively). Immature TSSM mortality was significantly higher on PAW1 (52%) and PAW2 (26%) treatments compared to control (6%). Immature developmental duration, adult pre-oviposition period and total pre-oviposition periods, adult longevity, fecundity, and sex ratio were all significantly reduced in response to PAW irrigation. Life table analyses showed that intrinsic rate of increase (*r*), net reproductive rate (*R*_0_), and finite rate of increase (*λ*) were significantly reduced on leaf discs from PAW-irrigated plants compared to control. Population modeling over a 60-day time showed a 10-11-fold reduction in TSSM populations on PAW-irrigated plants compared to control. These findings confirm the suppressive effects of supplementary PAW irrigation on TSSM population dynamics. Furthermore, results support the hypothesis that early-stage susceptibility, prolonged developmental times of individual life stages, and reduced fecundity are key factors driving PAW-based suppression of TSSM population dynamics. Thus, we conclude that supplementary PAW irrigation should be considered a potential component of long-term and sustainable pest management against TSSM and other major crop pests.

## Introduction

Two-spotted spider mites (TSSM) [*Tetranychus urticae* Koch (Acari: Tetranychidae)] are highly destructive agricultural pests^1,2^ with a host range of 1,100 known plant species^3^. In greenhouse tomato (*Solanum lycopersicum* L.) production, yield losses may be as high as 50% due to feeding causing chlorosis, reduced photosynthesis, and stunted growth^4,5^. Under severe infestations, TSSM produce silk-like webbing on host plants^6^. Additionally, TSSM infestations may induce stress, which increases susceptibility to secondary pests and diseases^7^. Traditional control methods often rely on frequent applications of synthetic acaricides^8^. However, rapid development of resistance in TSSM populations due to their short life cycle and high reproductive rate has rendered many acaricides ineffective^8,9^. Resistance development of this herbivore to 96 active ingredients has been reported (APRD, www.pesticideresistance.org/). Furthermore, the overuse of chemical agents poses serious environmental and health risks, highlighting the need for alternative, eco-friendly pest management strategies.

Releases of predatory mites, such as, *Phytoseiulus persimilis* [Athias-Henriot (Acari: Phytoseiidae)], have shown promise in management of TSSM populations^2,10^. However, the adoption of biological control programs in commercial tomato production has been limited due to inconsistent performance and due to practical constraints associated with needs for extensive pest population monitoring^11^. In certain tomato cultivars, this inconsistency is attributed to dense glandular trichomes, which can hinder the movement, establishment, and effectiveness of predatory mites^12,13^. Integrated Pest Management (IPM) strategies combining soft miticides with biological control agents have been recommended^14–16^. However, they may not be effective under high TSSM-pressure conditions, necessitating the development of preventive management tactics to minimize risks of high TSSM population densities.

High-voltage discharge to ionize gases at low or atmospheric pressure converting them into plasma has been known since the early 1900s^17–20^. This technology has been widely applied in surface sterilization of electronics, medical sterilization, fusion energy research, spacecraft propulsion, and biomaterials/food treatment^21,22^. Within the last decade, its applications have expanded, as it has been used to generate Plasma-Activated Water (PAW)^23,24^. PAW can be produced by introducing cold plasma ions from atmospheric air or gases like argon, oxygen, helium, or nitrogen into water^25–27^. PAW is rich in reactive oxygen and nitrogen species (RONS), including hydrogen peroxide (H₂O₂), nitrate (NO₃⁻), and nitrite (NO₂⁻), which offer multiple benefits, including in crop production. For instance, PAW has been shown to enhance seed germination, plant growth, nutrient uptake, and nutrient solubility^26,28–33^. PAW has also been shown to increase antioxidant enzyme activity and improve plant defense responses^34^. Additionally, it has been shown to suppress plant pathogenic viruses, fungi, and bacteria^35–37^. Finally, there is report of PAW possessing insecticidal activity against citrus mealybugs [*Planococcus citri* (Risso) (Hemiptera: Pseudococcidae)]^38^.

Our recent study investigated effects of PAW when applied as supplementary irrigation of tomato plants and its potential impact on TSSM populations^32^. Results demonstrated that supplementary PAW irrigation induced significant changes in leaf element composition and both non-glandular and glandular trichome densities, key components of plant resistance mechanisms against arthropod pests. These trichomes act as physical and chemical barriers, secreting high-viscosity allelochemicals such as acyl sugars, methyl-ketones, and sesquiterpenes that deter arthropod settling and feeding^39,40^. PAW-irrigated plants also caused a significant reduction in TSSM settling and overall population dynamics^32^.

These findings suggest that PAW can enhance plant defenses and indirectly suppress pest populations, offering a novel and eco-friendly approach to long-term and sustainable management of this and other important greenhouse pests. However, limited knowledge is available about effects of PAW irrigation of host plants on relative susceptibility of different pest life stages. A comprehensive breakdown of population structure, including sex and stage differentiation, is essential for informed decision-making in TSSM management programs^41,42^. Life table studies, which are powerful tools in population ecology, provide valuable insights by analyzing survival, development, and reproductive rates across specific life stages, linking individual-level responses to population-level outcomes^43–45^. Such approaches have been widely used to identify life stages or population structures that drive overall population dynamics under various stressors, including environmental conditions^46,47^ host plant quality^48–50^, and chemical treatments^51,52^. The age-stage, two-sex life table theory by Chi and Liu^53^ takes into account both sexes and stage-specific developmental rates among individuals. In so doing, it can provide comprehensive insight into population characteristics and be used to forecast temporal TSSM population dynamics in response to PAW irrigation^42,54,55^.

**Fig. 1:**
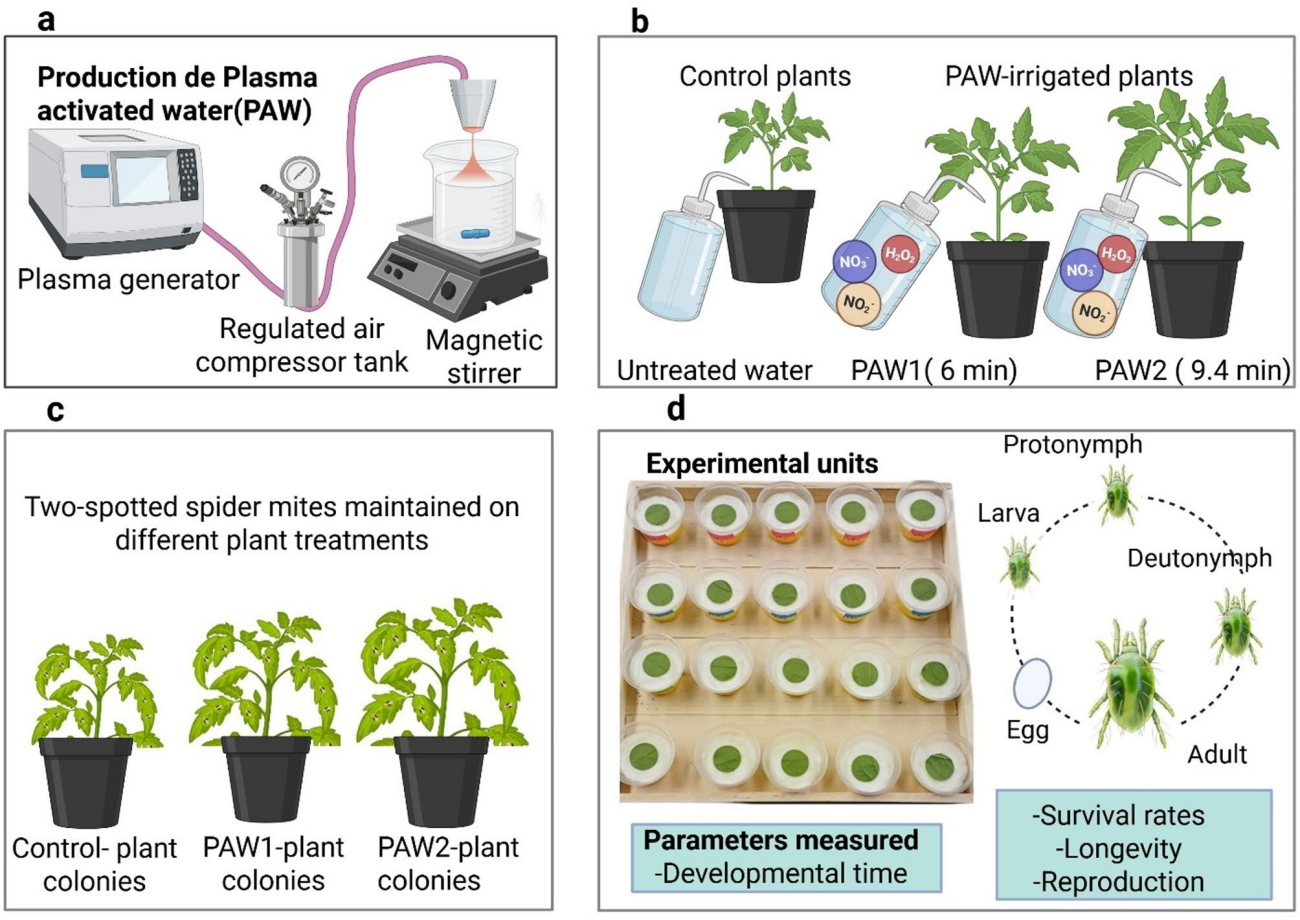
Overview of experimental setup and workflow. **(a)** Atmospheric plasma jet (APJ) used to generate PAW **(b)** Tomato plants irrigated with tap water (control) and with PAW1 or PAW2 (water treated for 6.0 and 9.4 min with APJ, respectively) **(c)** Colonies of two-spotted spider mite (TSSM) *Tetranychus urticae* on different experimental tomato plants **(d)** Experimental setup to evaluate effects of PAW-irrigated plants on TSSM life stages.

As outlined in Fig. 1, we examined the hypothesis that PAW irrigation of host plants has life-stage-specific effects on TSSM population dynamics. Results from this study are based on an analytical approach that has broad relevance to mechanistic analyses of demographic responses by arthropod populations to specific stressors. More specifically, in-depth insight into suppressive effects of supplementary PAW irrigation on TSSM population dynamics. Thus, we conclude that PAW irrigation should be considered a potential component of long-term and sustainable pest management tactics against TSSM and other major crop pests.

## Materials and methods

### Production and characterization of PAW

Two PAW regimes were used, where UC Davis tap water (control) was treated for either 6.0 min (PAW 1) or 9.4 min (PAW 2) and left for 24 h after treatment for further characterization. These specific durations were selected based on their distinct responsiveness in promoting plant growth observed in preliminary trials, including Savi, et al.^32^ which tested a broader range of PAW treatments. Physicochemical properties of PAW (pH, electrical conductivity, and oxidation-reduction potential) were measured using pH/EC(OHAUS-AB331M-F) and ORP meters (Hanna HI2202-01). Reactive species nitrate (NO₃⁻), nitrite (NO₂⁻), and hydrogen peroxide (H₂O₂) were quantified using a benchtop multiparameter photometer (HI83399-01). Measurements were collected following a completely randomized design with eight replicates per treatment, ensuring reliable data. PAWs were generated using an Openair™ Plasma System from Plasmatreat USA, Inc., as described in Savi, et al.^32^. For each treatment, 1 L of water was placed in a 2.5-liter Pyrex glass beaker and continuously stirred on a magnetic stirrer during plasma exposure (Fig. 1a). The setup ensured uniform treatment and prevented spillage with a corrosion-resistant steel cover. PAW samples used for supplementary irrigation of tomato plants were stored at 5 °C and were not older than four days at the time of application. Although literature reports that key reactive species such as H₂O₂, NO₃⁻, and NO₂⁻ can remain stable under cold storage for up to 21 days^56^ we deliberately limited the storage duration to four days to ensure the consistent use of freshly generated throughout the plant production cycle.

### Plants

Tomato cultivar Micro-Tom (*S. lycopersicum* L.) plants were used in the experiments. *Micro Tom* is widely recognized as a model cultivar in Solanaceae research due to its relatively small size and its short determinate life cycle^57^. This cultivar is also susceptible to herbivores including TSSM^13^ making it suitable for evaluating PAW effects on plant responses to TSSM. Micro-Tom seeds obtained from Seeds Buck (Augusta, California, USA) were grown in tray cells with a sterile, homogeneous substrate composed of pumice, sphagnum peat moss, sand, redwood sawdust, and dolomite. The growing medium was autoclaved at 121 °C for 1 h. Trays were placed in greenhouse facilities at the University of California, Davis, and an automated sprinkling irrigation system (Mix Rite injector, model 2502) provided water and fertilizer four times a day at 7 am, 10 am, 2 pm, and 5 pm. Fertilizer solution had the following composition (values in ppm): *N* = 150, *P* = 50, K = 200, Ca = 175, Mg = 55, S = 120, Fe = 2.5, Cu = 0.02, B = 0.5, Mn = 0.5, Mo = 0.01, and Zn = 0.05. Four weeks after sowing, seedlings were individually transplanted to 1.5-L pots 80% filled with a homogeneous mixture of soil, sand, and tanned bovine manure (1:1:1) autoclaved at 121 °C for 1 h. A 35 ml solution of water and fertilizer (prepared at the previously mentioned rates) was delivered to the pot twice daily (at 7 am and 6 pm) using an automated drip irrigation system. In addition, 50 ml of either PAW1, PAW2, or tap water was applied once a day, between 11 am and 12 noon, to each plant until the conclusion of the experiments (Fig. 1b). As a result, PAW treatments or control water accounted for 42% of the total water supplied to individual tomato plants.

Given that performance of arthropod herbivores is influenced by parental host plant (Maternal Effects Compensation Hypothesis) and that multiple generations are needed for physiological adjustment to a new host plant (Host Adaptation Hypothesis)^58,59^ TSSM colonies originally collected from soybean plants (*Glycine max* L. Merr.) at the Department of Entomology and Nematology, UC Davis, USA, were reared for several generations on control and PAW-irrigated plants before being used in experiments (Fig. 1c). Colonies were maintained in a greenhouse under controlled conditions: 25.2 ± 1.0 °C, 77 ± 10% RH, and a 12:12 L: D photoperiod.

### Experimental procedure

Experimental units consisted of plastic deli containers (50 mm diameter, 2 cm height) covered with a nylon foam layer (10 mm thick) lined with moistened cotton wool (Fig. 1d). A 30 mm diameter leaf disc from each treatment was placed abaxial side up in the center. Individual gravid TSSM females were transferred to leaf discs. After six hours, females and excess eggs were removed, leaving only one per disc. Using only individual eggs in experimental units is important to avoid bias induced by cannibalism and competition. Units were maintained under controlled conditions matching the TSSM colonies. Upon adulthood, TSSM individuals were sexed and paired in new units, with males from the stock colony used if needed. Units were examined daily under a stereomicroscope to record developmental stages, survivorship, size, mobility, and molting (via exuviae presence). Parameters recorded included pre-oviposition period (APOP: adult emergence to first oviposition), total pre-oviposition period (TPOP: first oviposition of parent to offspring), oviposition days, longevity, sex ratio, and fecundity. Males dying before females were replaced. Eggs laid were removed after every observation period, and leaf discs were replaced every three days to maintain leaf quality. Data on males from colonies and TSSM who died on the cotton wool strip while attempting to escape were excluded from statistical analyses.

### Data analysis

All data analyses were performed in R v3.6.1 software (R Foundation for Statistical Computing, Vienna, Austria). Physicochemical properties of PAW did not meet homoscedasticity and normality assumptions, even after transformations; they were analyzed based on nonparametric Kruskal–Wallis’ comparison of averages of treatments (library ‘AgroR’). Raw data on development, reproduction, and population parameters, and population parameters were estimated using an age-stage two-sex life table model^53^ developed by Chi^60^ and available at http://140.120.197.173/ecology/Download/Twosex-MSChart.rar. Parameters estimated (Table 1) included age-stage-specific survival rate (*s*_*xj*_), age-specific survival rate (*l*_*x*_), age-specific fecundity (*m*_*x*_), age-stage-specific fecundity (*f*_*xj*_), net reproduction rate (*R₀*), intrinsic rate of increase (*r*), finite rate of increase (*λ*), and average generation time (*T*). Standard errors (SE) of development, fecundity, reproduction period, and population parameters were calculated using a bootstrap procedure with 100,000 resamplings. Treatment differences were assessed using paired bootstrap tests (*B* = 100,000) at a 95% confidence interval^61,62^. TSSM population was projected using life table rate data based on the age-stage, two-sex life table theory^53,54^. Population growth over 60 days was simulated using the TIMING-MSChart software^42^with an initial population of 10 newly laid eggs across the three treatments.

**Table 1:** Equations used to estimate two-sex life table parameters.

**Age-stage-specific survival rate:** $\:{{S}}_{{x}{j}}=\frac{{{n}}_{{x}{j}}}{{{n}}_{01}}$ n01 is the total number of individuals used as the beginning of the life table study and nxj is the number of individuals surviving to age x and stage j.	(1)
**Age-specific survival rate:** $\:{l}_{x}={\sum\:}_{j=1}^{k}{s}_{xj}$	(2)
**Age-stage specific fecundity **(daily number of eggs per female of age x):$\:{{f}}_{{x}{j}}=\frac{{{E}}_{{x}{j}}}{{{n}}_{{x}{j}}}$ where Exj is the total eggs laid by individuals nxj.	(3)
**Age-specific fecundity** (average daily fecundity per individual, i.e., this number is divided by all individuals of age x):$\:{{m}}_{{x}}=\frac{{\sum\:}_{{j}=1}^{{k}}{{s}}_{{x}{j}}{{f}}_{{x}{j}}}{{\sum\:}_{{j}=1}^{{k}}{{s}}_{{x}{j}}}$	(4)
**Intrinsic rate of increase** was estimated by using the iterative bisection method of the Euler-Lotka equation:$\:{\sum\:}_{{i}={x}}^{{\omega\:}}{{e}}^{-{r}({i}+1)}{{l}}_{{x}}{{m}}_{{x}}=1$	(5)
**Finite rate of increase:** $\:\lambda\:={e}^{r}$	(6)
**Net reproductive rate**, defined as the total number of offspring an individual can produce during their lifetime:$\:{R}_{0}={\sum\:}_{x=0}^{\omega\:}{l}_{x}{m}_{x}$	(7)
**Mean length of a generation:** $\:T=\frac{{ln}{R}_{0}}{r}$	(8)

## Results

### Characteristics of PAW used for plant growing

**Fig. 2:**
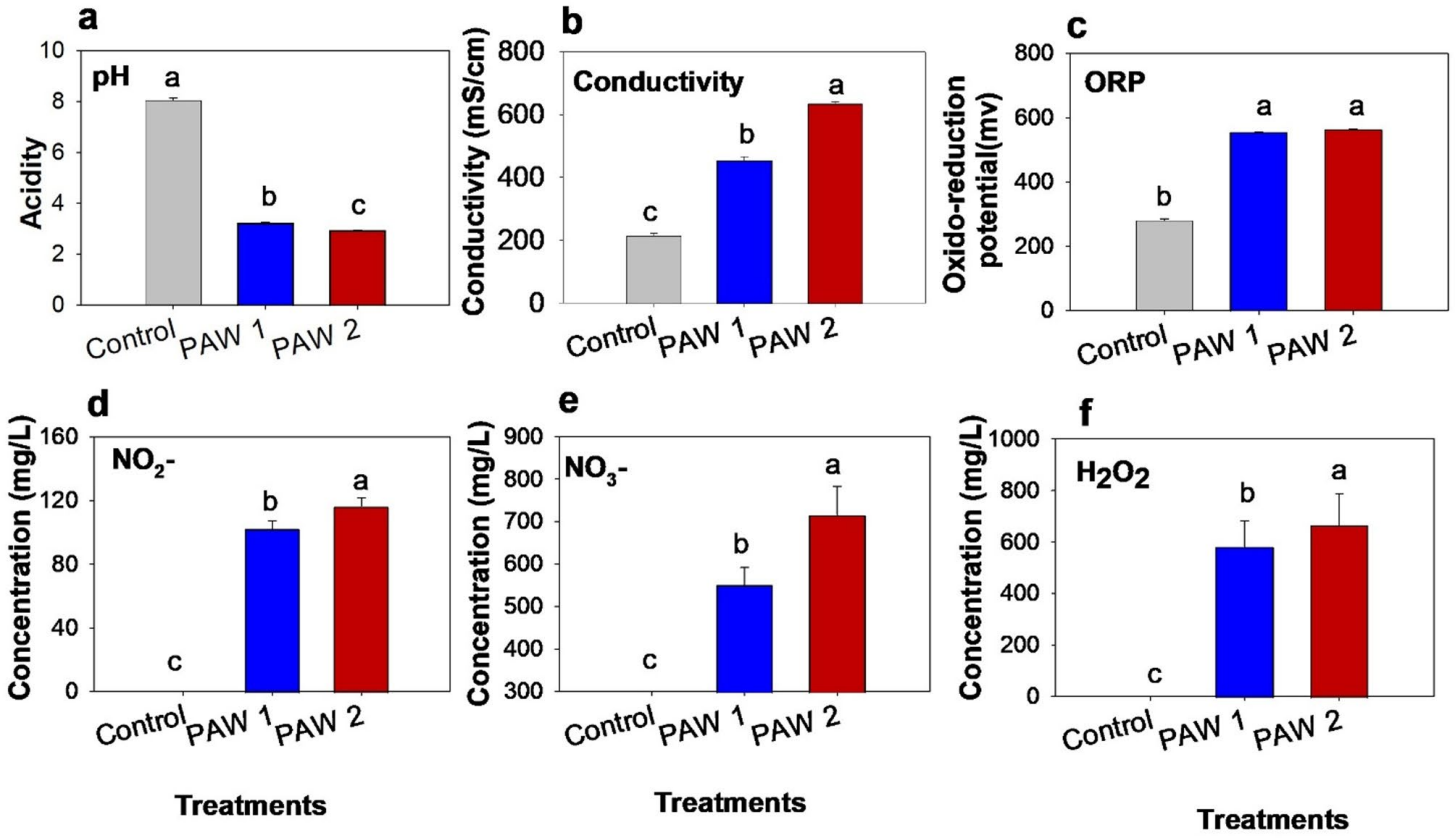
**(a)** pH, **(b)** Conductivity, **(c)** Oxidation–Reduction Potential (ORP), **(d)** Concentrations of Nitrate (NO_2_-, **e**) Nitrite (NO_3_-, and **f**) concentration of Hydrogen Peroxide (H_2_O_2_), in untreated tap water (control) and 6 min (PAW1) and 9.4 min (PAW2) treated plasma-activated water. Bars followed by different letters are significantly different (Kruskal–Wallis’s test, *p* < 0.05).

Compared to control, which had a pH of 8.8 ± 0.1, both PAW1 and PAW2 treatments showed significant reductions in pH (χ² = 23.19, df = 2, *p* < 0.001), with values dropping to 3.1 ± 0.1 and 2.9 ± 0.0, respectively (Fig. 2a). Electrical conductivity (EC) increased significantly (χ² = 23.14, df = 2, *p* < 0.001), with the highest value observed in PAW2 (633.8 ± 7.1 µS/cm), followed by PAW1 (454.1 ± 11.0 µS/cm), and the lowest in control water (214.9 ± 8.1 µS/cm) (Fig. 2b). A similar trend was seen in oxidation-reduction potential (ORP) (χ² = 23.16, df = 2, *p* < 0.001), with PAW2 having the highest value (563.2 ± 0.7 mV), PAW1 intermediate (553.8 ± 1.7 mV), and the control the lowest (281.4 ± 3.9 mV) (Fig. 2c). Nitrite (NO₂⁻) and nitrate (NO₃⁻) concentrations also increased significantly (NO₂⁻: χ² = 23.67, df = 2, *p* < 0.001; NO₃⁻: χ² = 23.15, df = 2, *p* < 0.001), with the highest levels in PAW2 (116.44 ± 5.38 mg/L and 716.55 ± 66.61 mg/L, respectively), followed by PAW1 (102.44 ± 4.87 mg/L and 551.55 ± 39.79 mg/L), and the lowest in the control (0.00 ± 0.00 mg/L and 0.77 ± 0.49 mg/L) (Fig. 2d and e). Hydrogen peroxide (H₂O₂) concentrations increased significantly in both PAW1 and PAW2 treatments, reaching 583.3 ± 98.3 mg/L and 666.7 ± 121.1 mg/L, respectively (χ² = 12.3, df = 2, *p* = 0.002), compared to the control (0.4 ± 0.3 mg/L) (Fig. 2f).

### Developmental stages, reproduction, and life table parameters

TSSM immature mortality rate was significantly higher on leaf discs from both PAW1- (52%) and PAW2- (26%) irrigated plants than control (6%). PAW1 led to the highest mortality rate (Table 2). Mortality rates varied across developmental stages. In PAW1 treatment, the highest mortality was observed in the egg (20%), larva (14%), and deutonymph (8%) stages. In PAW2, the highest mortality rate was observed in both egg and larval stages (12% for each stage). In contrast, the control group did not show mortality above 2% in any immature stage. No significant differences were observed in development times (in days) across treatments: eggs (8.0–8.1), larvae (3.1), and deutonymphs (3.1–3.4). Protonymph life stage varied significantly across treatments, with control showing the shortest duration (2.1), followed by PAW1 treatment (2.2 days), and PAW2 treatment eliciting the longest duration (2.4 days). TSSM female pre-adult time varied significantly among treatments, with PAW1 (17.0) having the most extended duration followed by PAW2 (16.7), while the control (16.4) had the shortest development time. TSSM male pre-adult time was significantly longer on leaf discs from PAW2-irrigated plants (16.8) than on leaf discs from PAW1-irrigated plants (16.0) and control (15.5).

Adult pre-oviposition period (APOP) was significantly longer on leaf discs from PAW1 (1.6 days) and PAW2 (1.7 days) irrigated plants compared to control (1.5 days) (Table 3). Similarly, total pre-oviposition period (TPOP) was significantly extended on leaf discs from PAW1- (18.6 days) and PAW2- (18.3 days) irrigated plants than on leaf discs from control (17.8 days). Oviposition days were significantly reduced in PAW2 treatment (9.9 days) compared to the control (13.0 days), with PAW1 treatment (10.5 days) showing an intermediate effect. Female adult longevity was significantly shorter on leaf discs from PAW1 (14.8 days) and PAW2 (13.6 days) irrigated plants than on control (17.3 days). Similarly, male adult longevity was significantly reduced on leaf discs from PAW1 (9.1 days) and PAW2 (8.0 days) compared to the control (14.2 days). Sex ratio (♀/♂+♀) was significantly lower on leaf discs from PAW1- (0.42) and PAW2- (0.50) irrigated plants than on control (0.82). Fecundity followed the same trend, being significantly reduced on leaf discs from PAW1- (37.14 eggs/female) and PAW2- (32.52 eggs/female) irrigated plants compared to leaf discs from control plants (49.93 eggs/female).

PAW treatments significantly affected life table parameters (Table 4). The net reproductive rate (*R*_0_) was significantly lower when plants were treated with PAW1 (15.60 offspring/individual) and PAW2 (16.26 offspring/individual) compared to the control (40.94 offspring/individual). Similarly, the intrinsic rate of increase (*r*) and finite rate of increase (*λ*) were significantly reduced in PAW1 (*r* = 0.113 day⁻¹; *λ* = 1.10 day⁻¹) and PAW2 (*r* = 0.115 day⁻¹; *λ* = 1.12 day⁻¹) relative to the control (*r* = 0.156 day⁻¹; *λ* = 1.16 day⁻¹). In contrast, the mean generation time (*T*) did not differ significantly among treatments (Control: 23.8 days; PAW1: 23.8 days; PAW2: 24.2 days).

### Age-survival rate and fecundity of the specific stage

**Fig. 3:**
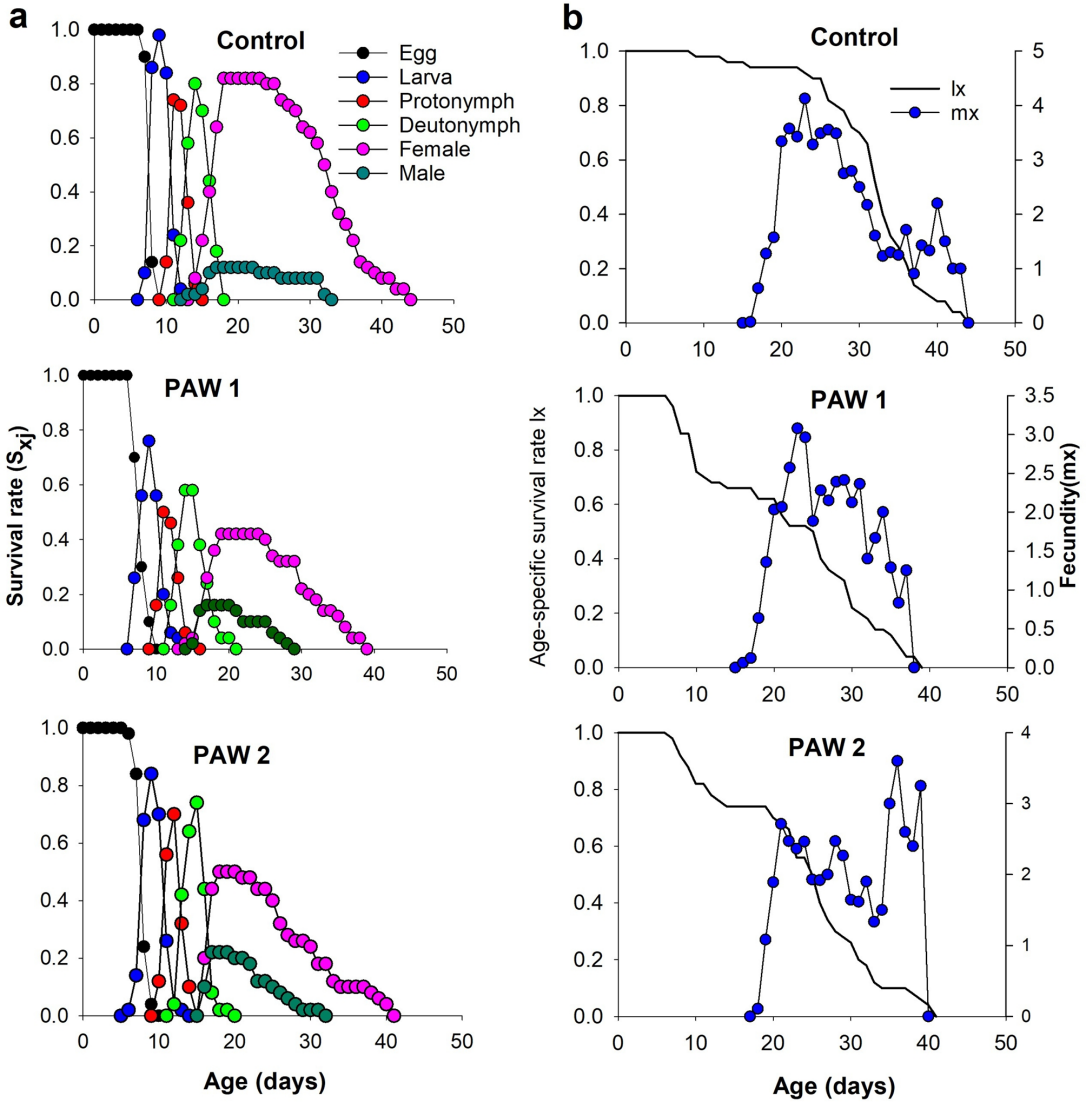
Variation in survival rates and fecundity of two-sppoted spider mite *Tetranychus urticae* across life stages on leaves from untreated and PAW-supplemented- tomato plants; **(a)** age-stage specific survival rates (*s*_*xj*_), and **(b)** age-specific survival rate (*l*_*x*_), age-specific fecundity (*m*_*x*_) and age-stage-specific fecundity (*f*_*x5*_). The two PAWs corresponded to plasma activation of water for 6.0 (PAW 1) and 9.4 min (PAW 2).

Age-stage survival (*s*_*xj*_) curves in Fig. 3a illustrate TSSM proportion at each developmental stage relative to the initial number of eggs (50) across different treatments. The observed overlapping proportions of distinct stages are due to variations in developmental rates among individuals. The proportions reached their maximum value, dropping as a function of molting to the next stage or mortality. The probability of a freshly laid TSSM egg surviving to the female adult stage was significantly lower on leaf discs from plants irrigated with PAW1 (0.42) and PAW2 (0.50) compared to the control (0.82) (Fig. 3a). In contrast, the probability of survival to the male adult stage was significantly higher on leaf discs from PAW1 (0.16) and PAW2 (0.20) treatments than on the control (0.12).

The age-specific survival rate (*l*_*x*_) represents the probability of a TSSM surviving up to age x across all developmental stages (Fig. 3b). Results showed that 80% of TSSM individuals maintained on leaf discs from PAW1- and PAW2-irrigated plants died before reaching 10 and 12 days of age, respectively. In contrast, those on control plants only experienced this level of mortality after 27 days. The age-specific fecundity (*m*_*x*_), defined as the average daily fecundity per individual at age *x* (Fig. 3b), showed the lowest peaks for PAW1 (3.07 eggs) and PAW2 (3.80 eggs) treatments on the 22nd and 20th days, respectively. In comparison, the control treatment exhibited the highest peak (4.12 eggs) on the 23rd day. Age-stage-specific fecundity *f*_*x5*_ (Fig. 3b), representing the daily number of eggs produced per female at age *x*, showed higher egg-laying peaks on control (4.73 eggs) on day 22 of their age. In contrast, peak fecundity *f*_*x5*_ was lower on PAW1 (3.80 eggs) and PAW2 (3.60 eggs) treatments on day 22 and day 35 of their age, respectively.

### *TSSM *population growth projection

**Fig. 4:**
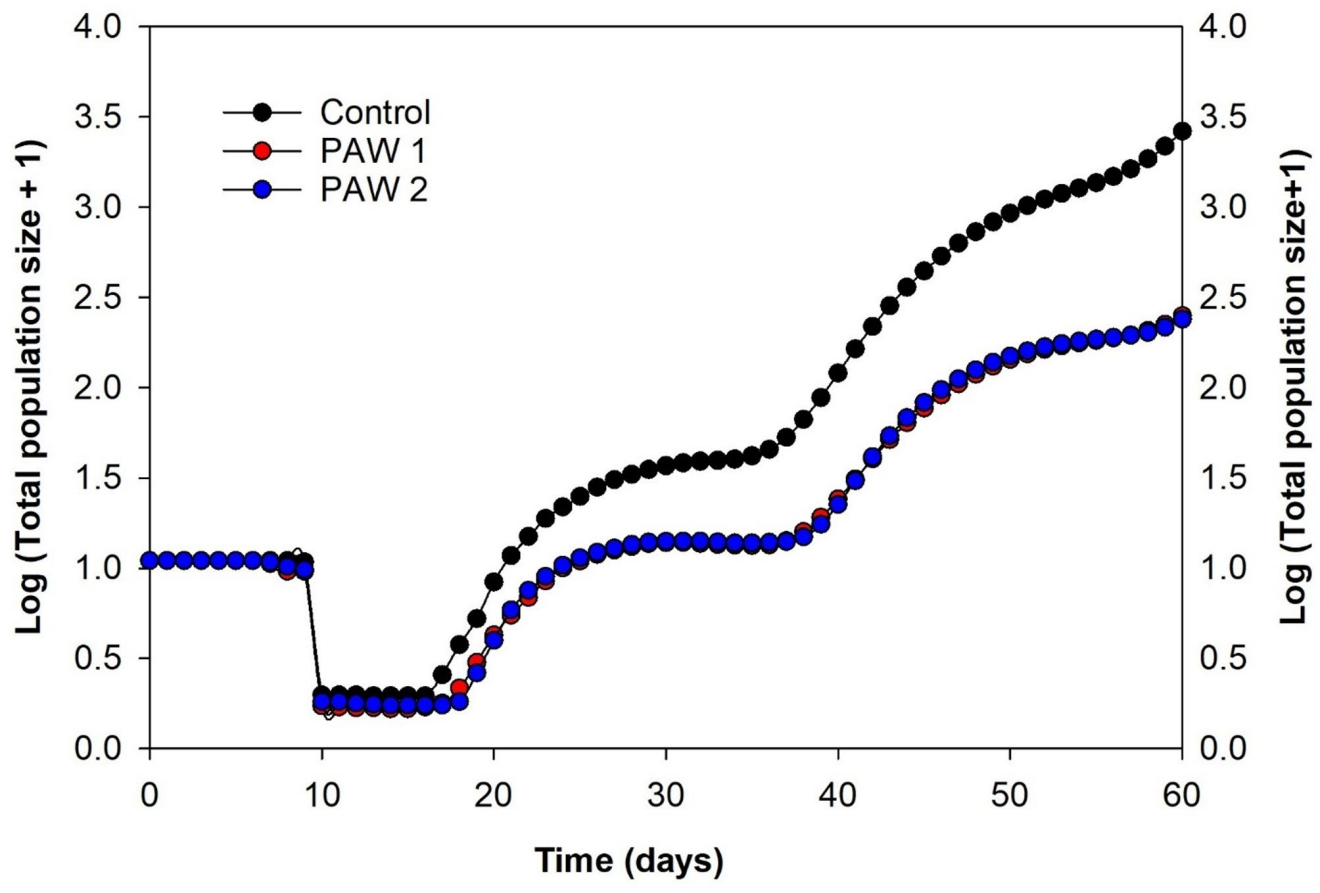
Sixty-day population projection of *TSSM* on leaves from untreated and PAW-supplemented- tomato plants from an initial cohort of 10 eggs. The two PAWs corresponded to plasma activation of water for 6.0 (PAW 1) and 9.4 min (PAW 2).

Figure 4 shows TSSM population projection over 60 days on control plants, PAW1- and PAW2-irrigated plants, starting from an initial cohort of 10 eggs. During the first 10 days, population size remained stable across treatments, as most individuals were still in the egg stage, resulting in minimal changes in population size. Between days 10 and 15, a decline was observed across all groups, likely due to molting events or early-stage mortality. From day 20 onward, the majority of TSSM reached the adult stage, leading to exponential population growth. By the end of 60 days, control treatment revealed the highest population size (2633.3 individuals). In contrast, PAW 1 (249.5 individuals) and PAW 2 (238.4 individuals) resulted in significantly lower population size, corresponding to an approximate 10.56- to 11-fold reduction in TSSM population compared to the control.

## Discussion

In a recent study, we demonstrated that supplementary PAW irrigation has potential to elicit significant TSSM avoidance and suppress population dynamics on tomato plants^32^. Pest population dynamics are largely driven by behavioral responses and relative susceptibility of individual pest life stages^63,64^so further insight into effects of supplementary PAW irrigation on individual life stages is needed. Accordingly, we performed two-sex life table analysis of TSSM when reared on plants subjected to supplementary PAW irrigation, and life table data were compared with those obtained from control plants. Our results revealed that supplementary PAW irrigation significantly increased mortality of immature TSSM life stages. Additionally, developmental durations and pre-reproductive phases were significantly prolonged in TSSM maintained on leaf discs from PAW-irrigated plants. We also demonstrated that supplementary PAW irrigation significantly reduced the proportion of females relative to males in a population (sex ratio), adult longevity, and fecundity compared to control. The intrinsic rate of increase (*r*), net reproductive rate (*R*_0_), and finite rate of increase (*λ*), which collectively reflect developmental, survival, and reproductive parameters, were significantly lower in TSSM populations maintained on leaf discs from PAW-irrigated plants. Moreover, a 60-day TSSM population projection showed a 10- to 11-fold reduction in PAW-irrigated plants compared to the control. These findings confirm the suppressive effects of PAW irrigation on TSSM population dynamics and support the hypothesis that early-stage susceptibility, developmental delays, and reduced fecundity are key factors driving this population dynamic. Consistent with our results, Dilip, et al.^65^ reported that rice plants grown from atmospheric cold plasma-treated seeds and irrigated with PAW elicited altered plant traits that negatively affected fall armyworm (FAW) [*Spodoptera frugiperda* (Lepidoptera: Noctuidae)]. They observed a significant reduction in larval mass gain and prolonged pupation periods, leading to a 25% increase in FAW mortality. These results suggest that PAW may broadly impact pest-plant interactions across various pest species, making it a valuable eco-friendly component of IPM strategies.

A major driver of PAW-driven suppression of TSSM population dynamics in PAW-irrigated plants appears to be the significant increase in immature mortality, which peaked at 52% in the PAW1 treatment and 26% in PAW2, in contrast to just 6% in the control. This suggests that significant physicochemical differences (i.e. pH, NO₂⁻, NO₃⁻, and H₂O₂) induced plant defense responses increased TSSM mortality. Previous studies have reported that leaf trichomes, when present in high densities in tomato plants, can elicit strong negative impacts on pest immature survival. These trichomes, categorized as non-glandular (types II, III, and V) and glandular (types I, IV, VI, and VII), act as physical and chemical barriers by secreting toxic exudates such as acyl sugars, methyl-ketones, and sesquiterpenes to impair pest movement and reduce survival^15,40,50,66,67^. Similarly, PAW irrigation has been reported to increase both glandular and non-glandular trichome densities, which may account for the higher mortality rates observed in PAW-irrigated plants^2,39^. This increase in trichome density is likely mediated by the high levels of ROS and RNS present in PAW. These ROS and RNS act as signaling molecules that influence hormone signaling pathways such as jasmonic acid (JA) and gibberellin (GA) known to trigger structural defense responses such as trichome proliferation and the activation of defense-related genes^68^ by modulating JAZ repressors or activating mitogen-activated protein kinase (MAPK) cascades^29,69^. At the molecular level, PAW irrigation also triggers oxidative stress responses, as reflected in the upregulation of miRNAs such as miR159, miR395, and miR398 each known to respond to H₂O₂ accumulation^30,70^. These miRNAs regulate key antioxidant enzymes and stress-related genes, contributing to sustained ROS signaling and enhanced defense readiness, including increased callose deposition and altered redox homeostasis^30^. Moreover, differences in H₂O₂ concentrations across PAW treatments likely influenced plant defense responses, contributing to variations in TSSM juvenile mortality. Although PAW2 contained a significantly higher H₂O₂ concentration than PAW1, it did not result in higher juvenile mortality (26% vs. 52%, respectively). This unexpected trend suggests a potential “optimal concentration effect,” in which moderate levels of H₂O₂ may be more effective at enhancing plant defenses than higher concentrations. Similar non-linear, dose-dependent responses have been reported in study by Adhikari, et al.^29^where excessive reactive oxygen species can lead to reduced efficacy due to compensatory detoxification mechanisms or hormonal imbalances.

A significant increase in development time was observed on leaf discs from PAW-irrigated plants. PAW1 treatment extended the development of individuals that matured into females, whereas PAW2 treatment caused a delay in those that matured into males. These findings suggest that PAW may interfere with TSSM molting and maturation depending on the quality of PAW applied. Furthermore, our results showed that PAW irrigation significantly reduced the proportion of females relative to males, adult longevity, and fecundity in TSSM populations, suggesting that supplementary PAW irrigation alters host plant quality in a way that affects sex allocation and reproductive success. Previous studies have shown that TSSM populations on tomato plants are typically female-biased, but this balance can be altered by extrinsic factors such as temperature^71^host plant quality^72,73^which is consistent with our findings. The shift toward a male-biased population on PAW-irrigated plants (PAW1: 0.42♀; PAW2: 0.50♀) compared to control (0.82♀) may be associated with reduced fertilization success, impaired sperm storage, or altered male reproductive behavior due to the high density of glandular trichomes and associated compounds, as previously mentioned^32^. Additionally, our visual observations indicated that eggs laid on leaf discs from PAW-irrigated plants appeared smaller than those on control plants, suggesting nutritional stress or limited resource availability. This is consistent with the mechanism described by Macke, et al.^74^who demonstrated that in TSSM, smaller eggs are less likely to be fertilized and more often develop into male offspring, whereas larger eggs are more likely to be fertilized and produce females. Thus, the observed reduction in female-to-male sex ratio, may be directly linked to egg size differences resulting from altered host plant quality. Therefore, the combined effects of a reduced female-to-male sex ratio and delayed reproduction (APOP) could contribute to reducing fecundity and slowing TSSM population expansion.

Life table parameters are widely recognized as reliable tools for assessing the bottom-up effects of host plants on pest populations^42,50,53,62^ Among these, the intrinsic rate of increase (*r*) is the most informative measure, as it integrates development, survival, fecundity, and sex ratio, serving as a key indicator of population performance^45,75,76^. In this study, *r* ranged from 0.156 day⁻¹ in control to 0.115 day⁻¹ in PAW-irrigated plants, falling within the reported range for TSSM populations on tomato varieties (0.060 to 0.295 day⁻¹)^72,77,78^. The significantly lower *r* in PAW-irrigated plants confirms its suppressive effect on TSSM population dynamics, making PAW irrigation a promising component for enhancing IPM strategies.

The 60-day population projection revealed around 11-fold reduction in TSSM populations on PAW-treated plants compared to the control. These results are consistent with recent findings under greenhouse conditions, where PAW-irrigated plants maintained consistently lower TSSM populations over three weeks^32^.This significant suppression suggests that PAW irrigation can serve as a long-term component for mitigating TSSM outbreaks, particularly in greenhouse environments where TSSM can rapidly reach economic injury levels. Additionally, delayed population growth has important IPM implications, as it can create a window of opportunity for natural enemies, such as predatory mites, to establish and control TSSM populations before they reach damaging levels.

In conclusion, this study provides strong evidence that increased immature mortality, delayed development, and reduced reproductive success are key drivers of the suppressive effects of supplementary PAW irrigation on overall TSSM population dynamics. Life table analyses have shown to be a crucial tool in assessing these impacts, reinforcing PAW’s potential as an effective component of IPM strategies. The ability of supplementary PAW irrigation to disrupt TSSM population dynamics suggests its potential to complement existing IPM tactics by reducing initial pest infestations and may favor predatory mites as biocontrol agents to suppress the TSSM population efficiently. Although studies of PAW-irrigated plant compatibility with biocontrol agents are still scarce, a significant increase in both non-glandular and glandular trichome densities by PAW in tomato plants^32^ may hinder the establishment of predatory mites. Additionally, a recent study investigated PAW direct effects on three species of entomopathogenic nematodes (EPNs) such as *Steinernema feltiae* Filipjev, *S. carpocapsae* Weiser, and *Heterorhabditis bacteriophora* Poinar^79^. Their results revealed PAW compatibility with *S. carpocapsae* but significant harmful effects on other two species. Therefore, further research is needed to evaluate PAW’s effects on a broader range of non-target organisms before its widespread implementation as a component of IPM programs. Further research is necessary to validate these findings on other crops and evaluate the long-term feasibility of PAW irrigation in diverse cropping systems. This will contribute to a deeper understanding of PAW-irrigated plant-pest interactions and support the development of sustainable pest management practices that enhance crop resilience while promoting ecological balance in agricultural systems.

**Table 2:** Mortality rate and mean (± SE) duration in days of the immature stages of two-spotted spider mite on leaves from untreated and treated-PAW tomato plants.

Treatment	*n*	Control	*n*	PAW 1	*n*	PAW 2
Immature mortality rate	50	0.06 ± 0.03c	50	0.52 ± 0.07a	50	0.26 ± 0.06b
Egg	49	8.02 ± 0.31a(2)	40	8.0 ± 0.15a (20)	44	8.1 ± 0.10a (12)
Larva	49	3.12 ± 0.16a (0)	33	3.12 ± 0.17a (14)	38	3.1 ± 0.12a (12)
Protonymph	48	2.06 ± 0.13b (1)	33	2.21 ± 0.10ab (0)	37	2.40 ± 0.11a (2)
Deutonymph	47	3.06 ± 0.21a (1)	29	3.44 ± 0.17a (8)	37	3.24 ± 0.14a (0)
Female pre-adult time	41	16.36 ± 0.65b	21	17.04 ± 0.34a	25	16.72 ± 0.13ab
Male pre-adult time	6	15.5 ± 0.82b	8	16.0 ± 0.25b	12	16.83 ± 0.32a

Numbers in bracts: percentage of dead specimens. Means within a row followed by the same letter are not significantly different. SEs were estimated using 100,000 bootstraps, and means were compared using a paired bootstrap test at 5% significance level.

**Table 3:** Mortality rate and mean (± SE) duration in days of the immature stages of Tetranychus urticae on leaves from control and PAW-irrigated tomato plants.

Treatment	*n*	Control	*n*	PAW 1	*n*	PAW 2
APOP	41	1.48 ± 0.09b	21	1.57 ± 0.18a	24	1.66 ± 0.12a
TPOP	41	17.85 ± 0.2b	21	18.61 ± 0.34a	24	18.34 ± 0.13a
Oviposition days	41	12.97 ± 0.76b	21	10.47 ± 1.11ab	24	9.87 ± 1.13a
Female adult longevity	41	17.29 ± 1.04a	21	14.76 ± 1.05b	25	13.6 ± 1.18b
Male adult longevity	6	14.16 ± 2.04a	8	9.12 ± 1.06b	12	8.0 ± 1.14b
Sex ratio (♀/♂+♀)	50	0.82 ± 0.05a	50	0.42 ± 0.09b	50	0.5 ± 0.07b
Fecundity(eggs/female)	41	49.93 ± 3.6 a	21	37.14 ± 4.83b	25	32.52 ± 4.51b

Means within a row followed by the same letter are not significantly different. SEs were estimated by using 100,000 bootstraps and means were compared by using paired bootstrap test at 5% significance level.

**Table 4:** Mean (± SE) duration of the pre-oviposition period (APOP), total pre-oviposition (TPOP), longevity, oviposition days, fecundity, and sex ratio of Tetranychus urticae reared on leaves from control and PAW-irrigated tomato plants.

Treatment	*n*	Control	*n*	PAW 1	*n*	PAW 2
*R*_*0*_ (Offspring/individual)	50	40.94 ± 4.28a	50	15.60 ± 3.28b	50	16.26 ± 3.19b
*r*(day^−1^)	50	0.156 ± 0.011a	50	0.113 ± 0.009b	50	0.115 ± 0.008b
*λ* (day^−1^)	50	1.16 ± 0.077a	50	1.10 ± 0.238 b	50	1.12 ± 0.008b
*T* (day)	50	23.78 ± 1.61a	50	23.81 ± 0.67a	50	24.15 ± 0.54 a

Means within a row followed by the same letter are not significantly different. The SEs were estimated by using 100,000 bootstraps and means were compared by using paired bootstrap test at 5% significance level. *R*_*0*_ = net reproductive rate; *r* = intrinsic rate of increase; *λ* = finite rate of increase; *T* = mean offspring time.

## Data Availability

The datasets used and/or analyzed during the current study are available from the corresponding author on reasonable request.
